# Phytoplankton growth regulation by dissolved P and mortality regulation by endogenous cell death over 35 years of P control in a Mountain Lake

**DOI:** 10.1093/plankt/fbab084

**Published:** 2021-12-22

**Authors:** William M Lewis, Jennifer Roberson

**Keywords:** phytoplankton biomass control, algal community composition, endogenous cell mortality, oligotrophication, lake phosphorus loading

## Abstract

Dynamics of phytoplankton and phosphorus were quantified in Lake Dillon, Colorado, over 35 years of P control. The lake provides an example of early intervention for P enrichment rather than remediation of advanced eutrophication. Phosphorus control began with tertiary treatment of effluent, which caused a phytoplankton decline (8.1–4.5 μg L^−1^ chla); a second decline (4.6–2.5 μg L^−1^ chla) occurred later following replacement of failing septic systems. Results showed that bioavailable phosphorus (BAP) loading was the only significant correlate of phytoplankton biomass; total P loading was not significantly related to biomass measured as chlorophyll. Phytoplankton composition changed greatly over the study interval, even though there was no long-term trend in potential causes of phytoplankton abundance other than reduction in BAP. Gradual decline of BAP loading also appears to have been the cause of large, gradual changes in phytoplankton community composition. Factors typically assumed to control phytoplankton mortality accounted for only ~50% of phytoplankton biomass turnover; the balance of mortality appears to be accounted for by endogenous cell mortality.

## INTRODUCTION

**Fig. 1:**
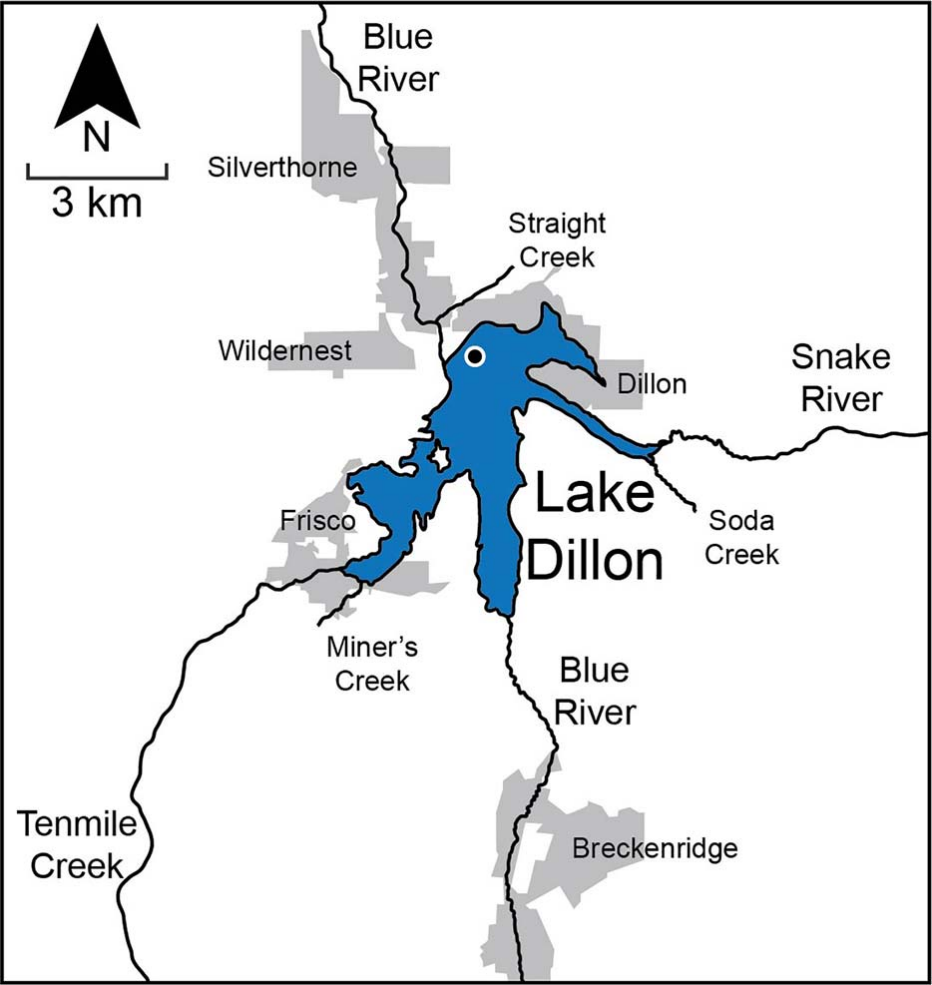
Map of Lake Dillon and surrounding areas. The circle indicates location of the index station, over deepest water. Copper Mountain resort is located on Tenmile Creek south of the map margin.

Lake Dillon was created in 1963 as a water supply for the City of Denver by impoundment of the Blue River near the US Continental Divide in Summit County, Colorado (volume at spillway level 0.317 km^3^, elevation 2748 m amsl, area 13.35 km^2^, mean depth 24.1 m, maximum depth 67 m, watershed area 85,160 ha; Lewis *et al*., 1984). The lake stores water from the Blue River and two of its tributaries, the Snake River and Tenmile Creek (Fig. 1). The headwaters for the three rivers reach the Continental Divide at a maximum elevation near 4300 m amsl.

Water is conveyed from Lake Dillon to Denver through a transmountain tunnel with a withdrawal point ~53 m below the reservoir surface. Water that cannot be stored is released to the Blue River below the dam (~50 m below spillway level), which has a minimum conservation flow of 1.42 m^3^ s^−1^. In addition, small amounts of water pass over the spillway during the runoff season (June, July) in some years.

The watershed of Lake Dillon consists largely of coniferous forest but also includes extensive area above treeline and wetlands along the three rivers. Development of the watershed is based on the ski industry, which is associated with four small municipalities (Fig. 1).

The USEPA and the State of Colorado in 1983 designated Lake Dillon for special protection of water quality in recognition of its scenic character as well as its importance to the Denver water supply and the recreational value of the lake and its watershed. Water quality control was implemented through a phosphorus concentration standard for the upper water column of Lake Dillon (7.4 μg L^−1^ growing season mean total P) and an associated requirement for control of phosphorus export from the watershed by the use of a total loading limit corresponding to the status quo of loading in 1982 along with formal arrangements for the use of phosphorus recovery projects to generate a quantifiable phosphorus bank to be used in accommodating further development of the watershed. All wastewater treatment facilities in the watershed were required to use tertiary treatment for control of phosphorus and land use practices were adopted within the watershed for the purpose of controlling nonpoint source pollution of the reservoir by phosphorus. Phosphorus in detergents, although identified in 1998 within the USA for voluntary regulation, was not regulated in Colorado or in the Dillon watershed. In the Dillon watershed, P from treatment facilities was captured by tertiary treatment of P in wastewater and replacement of septic systems with treatment plant service.

The Lake Dillon watershed was experiencing rapid development when the water quality regulation was adopted in 1983; the resident population then was 19,000 and seasonal peak population was 85,000. Development continued subsequently, but at a slower pace (present resident population 30,000; seasonal 160,000). Continuous studies of the lake and watershed from 1981 to 2016 provide the basis for an analysis of the response of the lake to the phosphorus control regulation. The control regulation was intended to prevent the annual load of phosphorus for Lake Dillon from rising in response to population growth. The 35-year data record shows that control of P did result in stabilization of total P loading, which was expected to cause corresponding stabilization of algal biomass, but phytoplankton biomass continued to decline for decades after total P load was stabilized. The purpose of the present analysis is to use the long-term data record to test hypotheses that might explain an unexpected 20-year decline in phytoplankton biomass. The long record also provides a basis for analysis of other lake features including phytoplankton dynamics and changes in phytoplankton composition.

Reduction in nutrient loading of lakes, primarily focusing on P, has been extensively documented; results vary widely (Kalff, 2002; Anneville *et al*., 2005). Some lakes show excellent remediation (e.g. Lake Washington, Edmonson and Lehman, 1981), whereas most show partial or minimal recovery, especially where internal loading based on legacy phosphorus offsets watershed nutrient reduction (e.g. Jeppesen *et al.*, 2002). The Lake Dillon nutrient reduction case is unusual in that the lake was, prior to phosphorus control, only at the upper margin of oligotrophy (~8 μg/L chla) and showed a low degree of hypolimnetic oxygen deficiency (oxygen ≥4 mg/L). Therefore, Lake Dillon gives an example of early intervention for enrichment rather than remediation of advanced eutrophication.

The Lake Dillon studies raise three questions leading to hypotheses that can be tested with data on Lake Dillon: (i) Given that total P loads and concentrations in the lake did not decline significantly in 1984–2016, what caused the decline in phytoplankton biomass for that interval? Hypothesis: Temporal variance in total P masked a decline in BAP (bioavailable P) that caused a decline in phytoplankton biomass. (ii) Given that zooplankton grazing and hydraulic loss consumed only a small portion of primary production, what was the fate of phytoplankton biomass production? Hypothesis: Because known loss rates of phytoplankton cannot be accounted for by known causes, significant losses are explained by factors internal to cells. (iii) What explains the drastic shift in species composition of Lake Dillon phytoplankton over the 35-year interval? Hypothesis: Given lack of multidecadal change in other factors likely to cause algal losses, change in P availability may explain the observed complex changes in phytoplankton composition.

## METHODS

Lake Dillon and its tributary waters were studied continuously between 1981 and 2016, with the exception of 1983. Data collection for years 1981 and 1982 occurred before full implementation of water quality control regulations for phosphorus, which extended from 1984 to 2016. Collection of data on the lake occurred at four stations: one over deepest water near the dam (index station), and one at the mouth of each of the three arms of the lake. Water samples were collected with a 5 m integrating sampler (Lewis and Saunders, 1979) deployed at 5-m intervals over the entire water column; temperature, specific conductance and pH were measured at 1-m intervals with a calibrated sonde. The data showed no significant difference among stations for water quality variables. For present purposes, analysis is limited to the index station, which provides information on samples from the surface to a point (60 m) near the maximum depth of the lake (67 m).

Transparency was measured as Secchi depth and in some years also with a quantum sensor as vertical PAR extinction, *K*_t_, m^−1^; relationship between the two was determined by their concurrent use over a 2-year interval: *K*_t_ = 1.7/*z*_sd_. Chlorophyll a (chla) was determined by hot ethanol extraction followed by spectroscopy (Sartory and  Grobbelar, 1984; APHA, 2005). Quantification of molybdate reactive P (MRP, here given as SRP), was analyzed manually by a phosphomolybdate method (APHA, 2005) with a 10-cm beam pathlength, and total dissolved phosphorus (TDP) was determined by persulfate digestion or by pyrolysis (Solorzano and Sharp, 1980) followed by SRP analysis. Both SRP and TDP samples were filtered with glass fiber filters (≈0.7 μm; APHA, 2005). Particulate P was estimated by hot acid digestion of the filtride followed by SRP analysis (Valderrama, 1981). Dissolved organic carbon (DOC) concentrations were determined with a carbon analyzer (APHA, 2005). Total suspended solids were captured on tared glass fiber filters (pore size ≈0.7 μm) and weighed (APHA, 2005). Total dissolved nitrogen was determined by oxidative digestion followed by liquid ion chromatography analysis for nitrate (Valderrama, 1981; APHA, 2005). Particulate nitrogen was determined with flash combustion followed by mass spectroscopy (APHA, 2005). Nitrate was determined by liquid ion chromatography (APHA, 2005).

Elemental ratios of phytoplankton biomass can be used in estimating mass components that cannot be measured directly. Estimates of mass ratios for present purposes are from Reynolds (2006) (Section 1.5): P/C, 2.4%; C/total dry mass, 50%; chlorophyll/total dry mass, 1.0%. The estimates are central tendencies for a range of values *in situ*.

Phosphorus that can be assimilated directly by phytoplankton is referred to as bioavailable (BAP), which consists of SRP (soluble reactive phosphorus) and any portion of DOP (dissolved organic phosphorus) that can be converted to SRP on the cell surface by phosphatase (Jones, 1972; Boström *et al*., 1988; Rengefors *et al*., 2001). The conversion process can be important when SRP is strongly depleted (e.g.1 μg/L). The BAP component of DOP cannot be measured. For present purposes, SRP is recognized as the dominant component of BAP.

Phytoplankton were collected from 1992 to 2015 as integrated water samples from 0 to 5 m on all sampling dates except dates in 1996–1998. Phytoplankton abundance by species was determined by the use of an inverted microscope with Lugol’s solution stain. Counts included picoplankton by the use of maximum magnification for the light microscope (1400×). Zooplankton were sampled within the top 20 m with a tow net of 37 μm mesh and preserved in formalin. Species and developmental stages were counted by methods as given in Lewis and Saunders (1979). The efficiency of the net as established empirically (Lewis and Saunders, 1979) was estimated as 50%.

Primary production of phytoplankton was measured by the use of ^14^C as reported by Lewis *et al*. (1984) and Morris and Lewis (1992). For the mixed layer, supersaturation of dissolved oxygen was interpreted qualitatively as evidence of positive net photosynthesis, and subsaturation was interpreted as negative net photosynthesis.

Phytoplankton species abundance across time was classified graphically because trends in abundance are too irregular to be expressed as linear or nonlinear functions of time. Overall abundance across years was scored as common (present in samples on most dates) or secondary (absent in samples on most dates). Temporal patterns in abundance of species were designated as increasing, decreasing, no trend or consistently high. Changes in composition of the community were evaluated on the basis of species, genera and divisions. Application of functional group analysis (e.g. Reynolds *et al*., 2002; Salmaso and Padisak, 2007) was not attempted because it is not yet standardized.

**Fig. 2:**
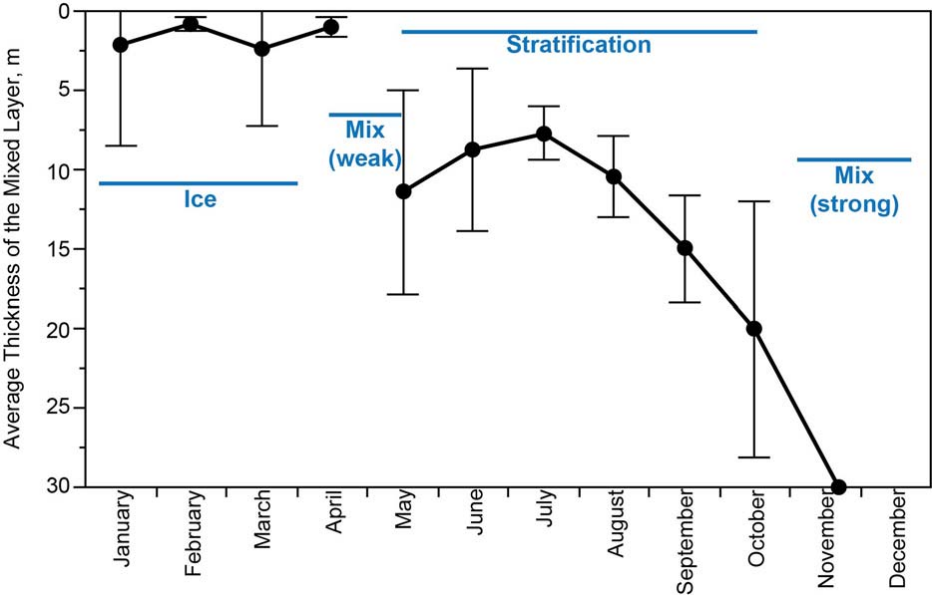
Monthly shift in mixed layer thickness over the stratification season (1981–2016, vertical bars show standard deviations).

Mass balance calculations for the lake were based on the use of a depth volume curve for determination of mass within specific intervals of depth and on daily data for water inflow and outflow as shown in records of the USGS, Bureau of Reclamation and Denver Water. Statistical analyses were conducted with JMP software.

The sampling schedule was designed to reflect differential rates of change in water quality and biological activity over the annual cycle (~15 times per year 1981–1999 and 6–15 times per year 2000–2016). Winter sampling was conducted primarily in January, when ice cover was suitable for lake sampling. In December, March and April, the lake was sampled less often than in other months because of the unreliability of ice cover.

Water entering the lake was monitored for volume and concentration of P and N as a means of estimating mass transport (loading) of P and N to the lake across months and year-to-year. Flow volume of the three rivers was determined by continuous data collection at gages operated by the USGS. Flow of the two streams entering the lake was determined by the use of current meters to create calibration curves relating flow to water depth at the points of entry for the streams. Depths were recorded for the two streams on each sampling date for P and N and used to compute flow for that date. For all five water sources, transport of P and N was estimated for each sampling date as the product of concentration and water flow. Sampling of flowing waters was done annually for the first 15 years and thereafter was done in alternate years. The time between sampling dates was adjusted to flow volume of the rivers; sampling was most frequent in the season of high flow.

## RESULTS

### Hydrology, temperature and mixing

Hydrologic information on Lake Dillon was derived from records of the Denver Water Department and from USGS gages on each of the three tributary rivers as well as estimates of contributions by smaller surface water sources, groundwater and precipitation (Lewis *et al*., 1984). The annual mean volume of Lake Dillon was 289 × 10^6^ m^3^; annual mean hydraulic residence time was 1.19 ± 0.29 years. Lake Dillon receives water primarily from snowmelt, which enters the lake largely as a dominant surge in discharge that begins in May and ends in July. Water exits the lake primarily from the hypolimnion.

Lake Dillon typically had ice cover between late December and April with considerable interannual variation and showed inverse stratification under ice cover (Fig. 2). Evidence of stability in layering began to appear in May, but warm season stratification was not well established until June. The mixed layer thickened monthly after July until late November, when autumn mixing occurred (Fig. 2).

The epilimnion lost water over the spillway in most years (27 of 35), but the loss rates were small. Maximum loss as % of mixed layer volume per day for June–October over the 35 years was 3.2, 3.4, 0.9, 0.3, 0.2.

The mixed layer of the lake warmed ~2.5°C over the 35-year study interval (Lewis *et al*., 2019). Deeper water warmed as well, but at a lower rate. Water column stability increased by 41%, but mean mixed layer thickness did not change, nor did the mean ice-free date for the lake (May 10, ±10 days). The low surface temperature of the upper water column during stratification caused greater short-term variation in thickness of the mixed layer than would occur for a similar lake at lower elevation. This type of variation could obscure a small long-term trend in thickness of the mixed layer that might result from warming.

### Transparency

**Fig. 3:**
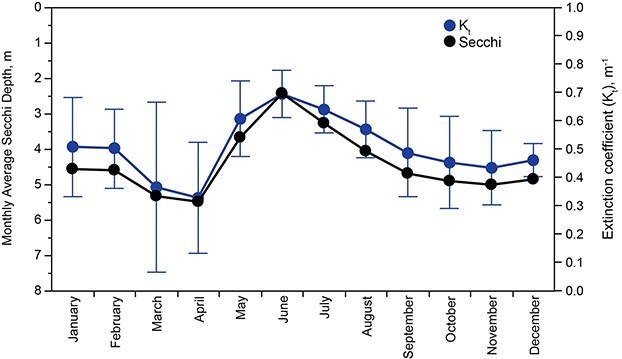
Monthly means and standard deviations for Secchi depth in Lake Dillon, 1981–2016, and corresponding values of total PAR extinction (Secchi depth, *K*_t)_.

Transparency of Lake Dillon as measured by Secchi depth (Fig. 3) showed no interannual trend across the 35-year study interval. Components of PAR (Table I) were quantified from concentrations of DOC, chla and total suspended solids by methods as described by Lewis (2011).

**Table I:** Mean proportionate contributions to *K*_t_ in Lake Dillon (mean chlorophyll for ice-free season, 4 μg/L)

Component		Value, m^−1^	SD
*K*_w_	Water	0.10	0^a^
*K*_g_	DOM	0.13	0^a^
*K*_a_	Algal biomass	0.066	0.035
*K*_p_	Nonliving particles	0.24	0.19
*K*_t_	Total	0.54	0.20

^a^Low variance, treated as a constant.

### Phosphorus

The data record for phosphorus concentrations consists of three phases. Phase 1, 1981–1984, includes 1981–1982, prior to control of P loading for the lake, and 1983–1984, when tertiary removal of P from municipal wastewater treatment plants was completed. In 1985–1995 (Phase 2), additional attempts to control P loading focused on soil disturbance and urban runoff. In Phase 3 (1996–2016), control of P load from failing septic systems was implemented gradually through installation of sewer lines that took domestic wastewater to tertiary treatment plants.

**Fig. 4:**
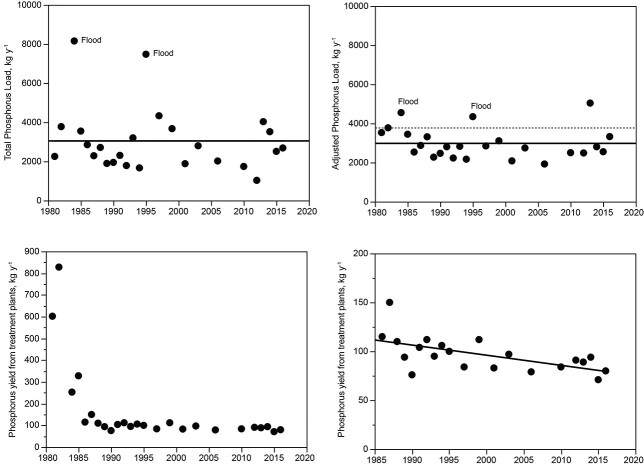
Above left: Total P load for Lake Dillon from all sources, 1981–2016. Above right: P load adjusted to 1982 hydrologic conditions. The solid line shows the mean; the dashed line is the 1982 benchmark for the control regulation (3778 kg year^−1^). Below left: Total P discharge from wastewater treatment facilities, 1981–2016, showing effect of tertiary treatment. Below right: Total P from treatment plant discharge after implementation of tertiary treatment (1986–2016) showing refinement of tertiary technology. A data point for 2008 is omitted because of anomalous P releases caused by a failure of one facility to maintain fully effective treatment over part of a year.

Linear regression showed no statistically detectable trend in total P over the 31 years after Phase 1 (Fig. 4,1985–2016, mean 2840 kg year^−1^, *P* > 0.05). Figure 4 also shows annual total load adjusted for variations in hydrology (mean 2880 kg year^−1^). The adjustment was accomplished through the use of a watershed model that included empirically based, calibrated relationships between phosphorus yield and runoff for specific source categories, including background, housing served by septic systems, housing served by sewer, ski resorts and others (Lewis *et al*., 1984). Modeling was used to adjust all loads to a common hydrologic year, 1982, the reference year for the control regulation. The use of the model reduces variance related to hydrology among years, which allows more accurate assessment of compliance with the control regulation. Years 1984 and 1995 showed anomalously high loads because of flooding. In 2013, recovery from drought caused an anomalous increase in particulate P transport caused by runoff over dry soils.

Figure 4 shows the effect of tertiary treatment in controlling phosphorus release from municipal treatment plants (Phase 1). The effect of tertiary treatment was strongest in 1981 and 1982, which showed a reduction in phosphorus release of ~700 kg year^−1^. Figure 4, right panel (expanded scale for 1986–2016), shows that additional improvements in efficiency of tertiary treatment after 1985 accounted for a further reduction of ~30 kg year^−1^ in total P.

There was no detectable trend in runoff-corrected annual loading of total P after implementation of tertiary treatment for P in 1985 (*P* > 0.05, Fig. 4, upper right panel). The rapid decrease in P loading (700 kg year^−1^) caused a sustained reduction in load to the lake, but the subsequent load reduction measures were not reflected in the measured load to the lake because they occurred gradually and therefore were subject to offset by incremental doubling of the population over the same interval. Restriction of soil disturbance effects (1985–1995) during Phase 2 had only modest potential to reduce total load, as shown by field studies of specific sites (Lewis *et al*., 1984). During Phase 3 (1996–2016), the effect of septic system removal, as estimated empirically through watershed mass balance studies (Lewis *et al*., 1984), had higher potential (~250 g P year^−1^ per septic system, watershed total ~200 kg year^−1^). The reduction of load from septic systems likely occurred gradually beyond the deactivation date of specific systems because of terrestrial migration of septic P that was distributed to streams. Overall, the phosphorus control measures between 1985 and 2016 were successful in preventing an increase that could have resulted from population growth.

**Fig. 5:**
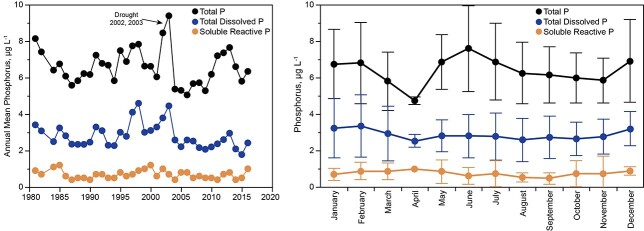
Left: Total P, TDP and SRP for Lake Dillon, 0–5 m, as annual means. Right: Concentrations of total P and P fractions at 0–5 m in Lake Dillon as monthly means and standard deviations for 1986–2016.

For 1981–2016, total phosphorus in the mixed layer (0–5 m) of Lake Dillon averaged 6.6 ± 1.0 μg L^−1^ (all months, Fig. 5). Mean concentrations of SRP and TDP for 1981–2016 were 0.7 ± 0.2 and 2.8 ± 0.7 μg L^−1^. A severe drought that drastically reduced the lake volume in 2002–2003 produced notable peaks in TP and particulate P that did not reflect increased loading from the watershed. The peaks were caused by wind, which mobilized sediments over the shallow water of the lake at drawdown. Total P showed no overall trend and no statistical relationship to total annual water inflow or hydraulic residence time for 1981–2016 (Fig. 5). Regression was not applied to SRP because its concentrations often equal or approach detection limits; DOP, which is calculated from SRP, also does not qualify for regression. This deficiency is of no practical importance because SRP is efficiently assimilated by phytoplankton, which means that its concentrations in lake water are not reflective of loading rates from the watershed.

Mean monthly concentrations of total P in the upper water column of Lake Dillon showed suppression in March and April, probably because of the addition of lake ice melt water to the upper water column at that time, and a peak in May–July during spring runoff caused by particulate P from the watershed that subsequently settled from the water column (Fig. 5). Variations in total P across months were weakly reflected or not reflected in TDP or SRP, i.e. variation in total P was caused mostly by particulate P.

**Fig. 6:**
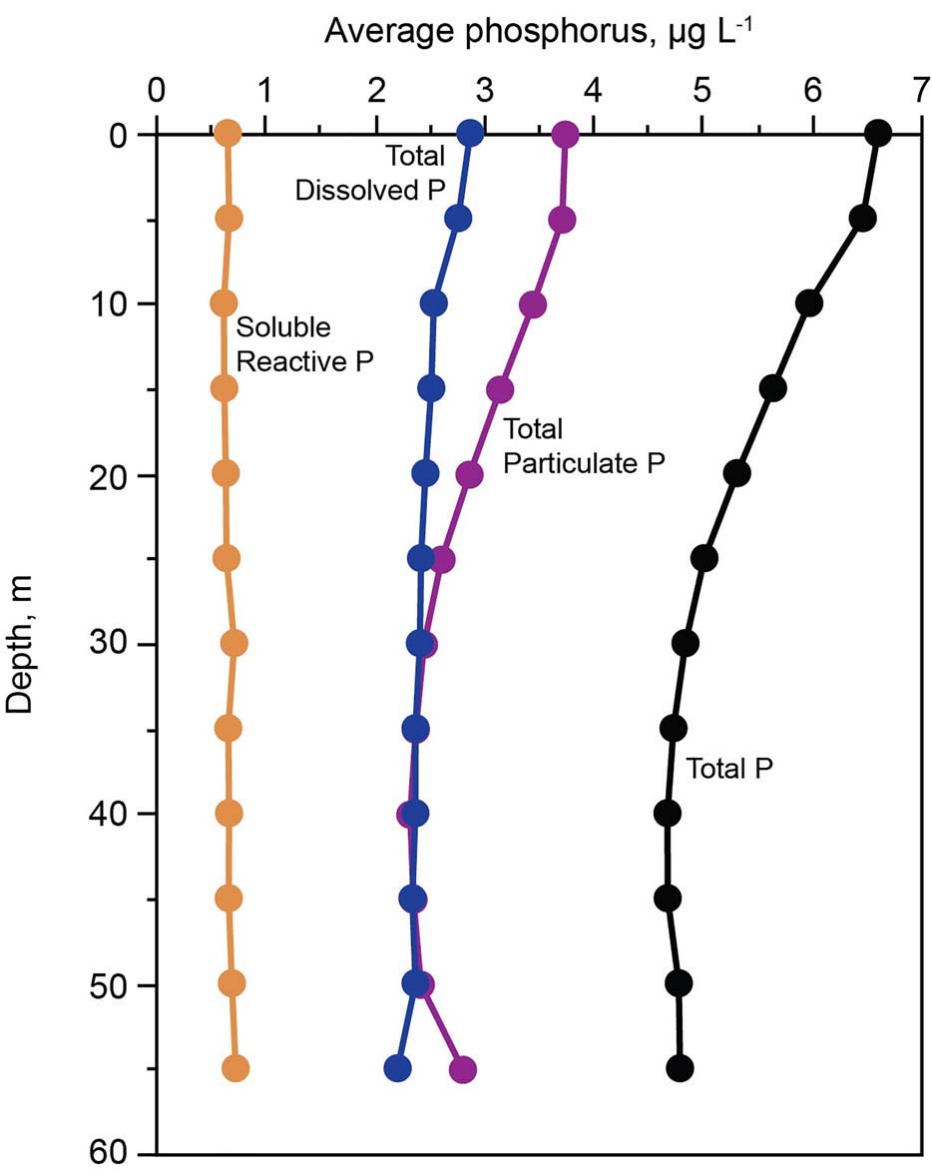
Average concentrations of phosphorus fractions across all depths, 1986–2016.

Vertical distributions of TDP and SRP were nearly uniform, but particulate P showed ~30% higher concentrations in the upper 10 m than at greater depths (Fig. 6). As shown by the ratio of chlorophyll and total P to dry algal biomass (see Methods section), the vertical pattern of particulate P was not caused by algal biomass, which was a small component of particulate P in most months, but rather by non-algal particles. Because the lowest water column concentrations of dissolved oxygen were above 4 mg/L, there was no redox-related acceleration of P release from deepwater sediments (Fig. 6). P release from sediments under oxic conditions is low in montane lakes of the study region (e.g. Anthony and Lewis, 2012).

For Phase 3 (1996–2016), data on individual watersheds showed potential for reduction of load, especially for SRP, the most mobile component from septic systems. Data on river loading confirmed this hypothesis (Table II).

**Table II:** SRP for Lake Dillon and its three river tributaries, given as a mean for 1996–2016, and degree of change over the same interval as determined from regression analysis

	Mean, μg L^−1^	Initial, μg L^−1^	Change, μg L^−1^	*r* ^2^	*P*
SRP					
Blue River	1.7	2.4	--1.3	0.69	<0.01
Snake River	1.2	1.7	--0.9	0.59	<0.01
Tenmile Creek	1.1	1.1	0.0	-	NS
Lake	0.7	0.7	0.0	-	NS

Significant decline in concentrations of SRP (Table II) occurred in the Blue River (54%) and in the Snake River (53%) at their entry points to Lake Dillon. There was no trend in SRP for Tenmile Creek, probably because the ratio of water discharge to septic sources of P for this watershed was too high to allow quantification of temporal change in SRP.

In the lake, concentrations of SRP for 1996–2016 did not decline in response to decreased loading of SRP, despite the decline in septic system sources of SRP. Because of SRP scarcity prior to the reduction in SRP loading, uptake by phytoplankton was occurring at maximum efficiency over the entire study interval, as shown by consistently very low SRP concentrations in the mixed layer during the growing season. Under these conditions, additional SRP from the watershed would be assimilated by phytoplankton and would not remain dissolved in the lake.

### Nitrogen

**Fig. 7:**
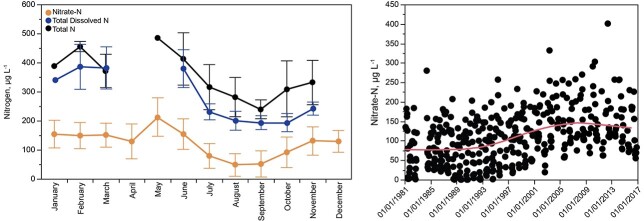
Left: Concentrations of total N (2011–2016), total dissolved N (2014–2016) and nitrate (1981–2016) in Lake Dillon (0–5 m), with means and standard deviations. Right: Temporal changes in nitrate concentrations for Lake Dillon (0–5 m, all dates), 1981–2016. The red line shows a LOWESS polynomial fit for the data.

Total nitrogen and dissolved nitrogen were analyzed only for a portion of the study interval; nitrate was analyzed for all sampling dates (Fig. 7). Concentrations of ammonia were measured infrequently they were consistently very low (typically <5 μg L^−1^), implicating nitrate as the main nitrogen supply for phytoplankton. Because nitrogen was not included in the special water quality regulations that were adopted for Dillon, nitrate was subject to influence by watershed development, whereas phosphorus was regulated as needed to maintain the status quo as of 1982.

Mean nitrate concentrations in the upper water column of Lake Dillon increased threefold between 1981 and 2009, in parallel with watershed development (Fig. 7), reflecting primarily the effects of wastewater treatment plants and septic systems (Kaushal *et al*., 2006). After 2009, the trend reversed (Fig. 7) in response to improved nitrogen removal from wastewater effluent and connection of septic systems to sewer.

Annual minimum concentrations for nitrate in the mixed layer of the lake prior to 1997 were often near zero in summer (Fig. 7). After 1997, there was a continuous increase in the annual minimum nitrate concentrations. Nitrate concentrations increased throughout the water column, but the mean concentrations of nitrate were higher at intermediate and great depths than near the surface. As the mixed layer thickened in autumn, its nitrate concentrations increased. Nitrate depletion near the surface coincided with favorable growing conditions for phytoplankton.

### Algal biomass (chlorophyll)

**Fig. 8:**
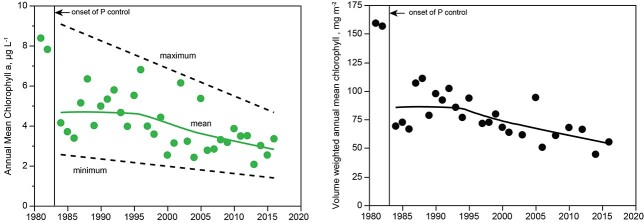
Left: Concentrations of chla in Lake Dillon, 0–5 m. Points show annual means; the solid line shows no trend for 1985–1995, and a significant decline thereafter (1996–2016, *r*^2^ = 0.28, *P* < 0.01). The dashed lines show the trends for annual maximum and minimum. The green line is a LOWESS polynomial regression (alpha = 0.75). Right: Annual mean chlorophyll per unit area of Lake Dillon, 1981–2016. The trend line is a LOWESS polynomial regression (alpha = 0.75). In year 2002, chlorophyll per unit area was greatly affected by drawdown of reservoir volume due to drought and is excluded from the regression (*r*^2^ = 0.37, *P* < 0.01).

For 1981–1982, prior to completion of tertiary treatment of P for wastewater, mean chlorophyll concentrations in the mixed layer (8.1 ± 3.7) were significantly greater than mean concentrations for 1984–2016 (4.1 ± 1.9), after tertiary treatment for P had been implemented (Fig. 8). Following the abrupt change in chlorophyll caused by tertiary treatment in 1981–1982, the interval between 1984 and 2016 showed no change until about 1996, after which there was a decline to 2016. The trend line for annual means corresponds to a decline from 5.1 to 2.9 μg L^−1^ from 1986 to 2016, i.e. a change of 43%. Chlorophyll a in the mixed layer showed no significant statistical relationship to hydraulic residence time or total annual water inflow to Lake Dillon over the study interval.

**Fig. 9:**
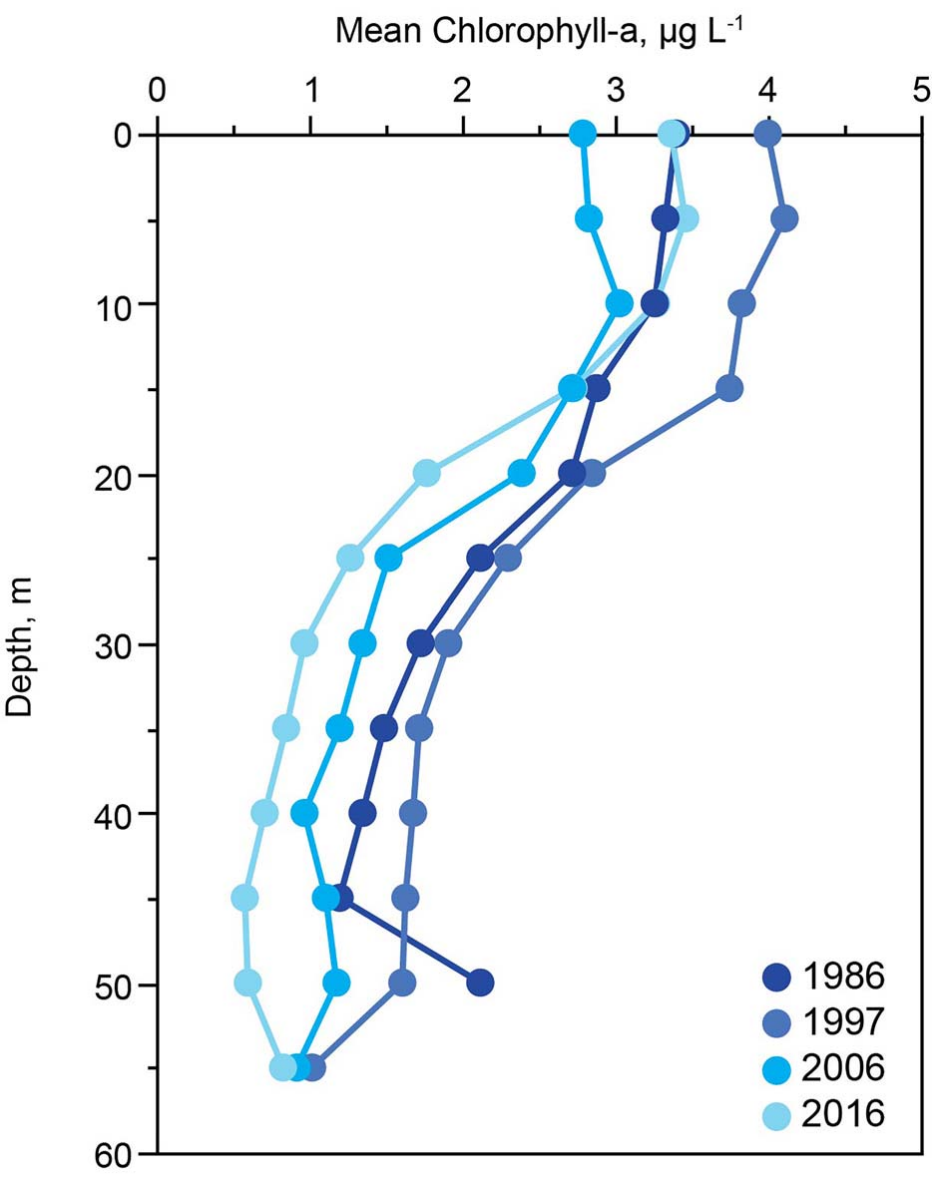
Mean annual chlorophyll with depth for specific years at decadal intervals.

Chlorophyll concentrations below the euphotic zone averaged ~30% of chlorophyll in the euphotic zone (Fig. 9) and showed high stability except in November–December, when deep mixing homogenized the concentrations vertically.

**Fig. 10:**
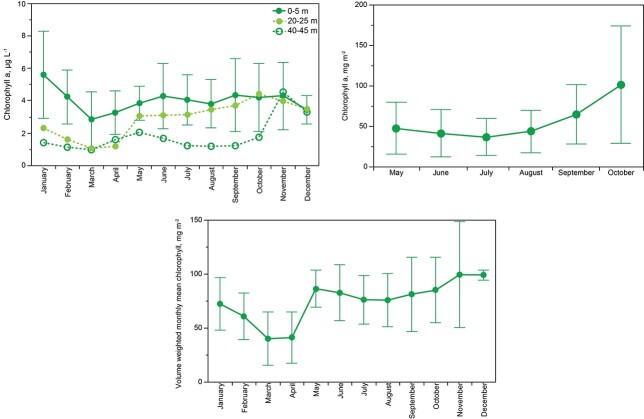
Top left: Abundance of chla (monthly means with standard deviations, 1984–2016) at 0–5 m, 20–25 m, 40–45 m. Top right: Chlorophyll per unit area for the mixed layer, monthly mean and standard deviations, 1984–2016. Bottom: Means and standard deviations for chlorophyll per unit area, full water column, for all months 1984–2016.

Across months near the surface (0–5 m), January showed the highest mean chlorophyll concentration (Fig. 10). March showed the lowest concentration of chlorophyll and the establishment of stratification (May) was accompanied by an increase in mean chlorophyll concentrations leading to nearly constant concentrations between June and November. Interannual variance for all months was considerable, as would be expected given interannual variance in meteorological conditions (cf. Finger *et al*., 2013).

Chlorophyll concentration per unit volume in the mixed layer (Fig. 10) showed no interannual trend in abundance per unit volume of phytoplankton over the stratification season (May to mid-November). Chlorophyll per unit area in the mixed layer (Fig. 10), however, showed a significant seasonal increase in phytoplankton biomass per unit area beginning in August and ending in November at the time of full mixing; growth corresponded to thickening of the mixed layer toward the end of the stratification interval as algae and nutrients below the thermocline entered the mixed layer. Although the chlorophyll per unit volume in the mixed layer was essentially constant, the amount of mass per unit area almost doubled as the thickness of the mixed layer increased. Under ice, chlorophyll was more abundant in the upper water column than the lower water column.

Figure 10 shows mean phytoplankton biomass per unit area for the entire water column over all months. November was the month of maximum chlorophyll per unit area, which carried into December with no change but then showed a loss of ~50% of water column chlorophyll from January through March, when abundances reached ~35 mg m^−2^, which was about a third of the maximum that was observed during November and December (Fig. 10). Net mortality of phytoplankton under ice cover was ~0.67 mg m^−2^ day^−1^, calculated as mean annual difference per unit area of the lake before and after full thickening of the ice cover. Stabilization of the mixed layer in May corresponded to a significant increase in net production of biomass. The May peak led to slight decline in August, after which accumulation occurred in response to thickening of the mixed layer.

### Photosynthesis, dissolved oxygen, respiration

**Fig. 11:**
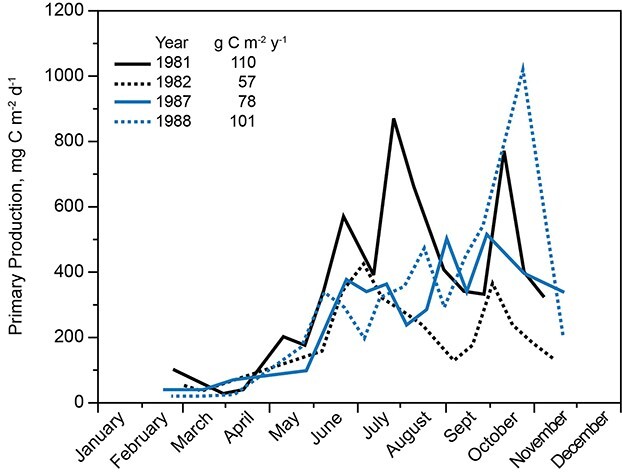
Net primary production per unit area in Lake Dillon for 4 years. Redrawn from Lewis et al. 1984, Morris and Lewis 1992.

Measurements over 4 years show that net primary production was low under ice and during ice melt until May, when water column stabilization began (Fig. 11). Net production averaged ~400 mg C m^−2^ day^−1^ between the middle of May and the end of October, but production showed some depression between the middle of August and the middle of September. As the mixed layer began thickening in September, photosynthesis per unit area increased until mixing depth exceeded 20 m (October), then declined as the lake moved toward full mixing.

**Fig. 12:**
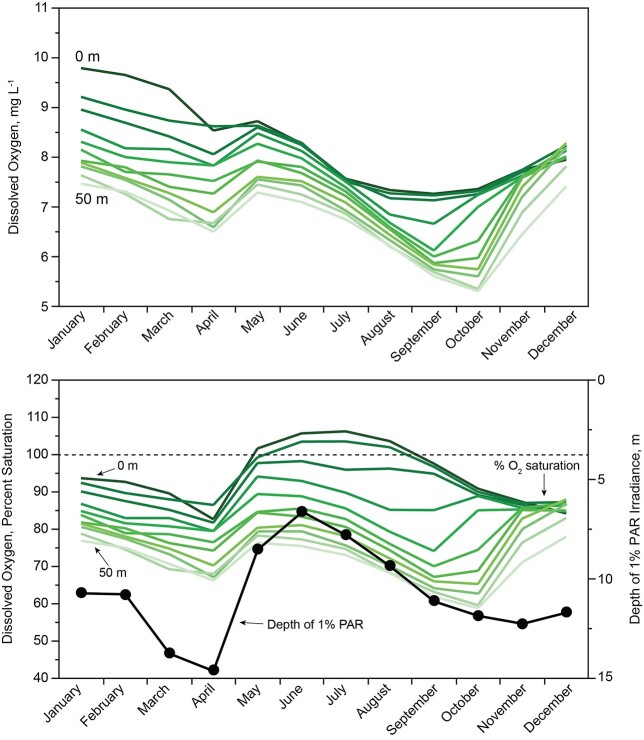
Above: Mean dissolved oxygen concentrations by depth (1984–2016). Below: Monthly mean % saturation of dissolved oxygen for 1984–2016 at 5 m increments from 0 to 50 m in Lake Dillon, and mean depth of 1% surface PAR over the same interval through a hole in the ice cover (January–April) and for the ice-free water column (May–December).

Mean minimum interannual oxygen concentrations for the hypolimnion were above 5 mg L^−1^ (Fig. 12); for individual dates across all years, the minimum was near 4 mg L^−1^. Under early ice cover, from the middle of December through January, the upper water column accumulated oxygen through photosynthesis at 0–15 m, but lost oxygen below 15 m (Fig. 12). Later, under ice, in February and March, concentrations of dissolved oxygen declined at all depths in synchrony with snow accumulation over the ice.

The lake was undersaturated with oxygen at all depths during autumn mixing and under ice (Fig. 12). Upward movement of percent saturation in April coincided with reaeration immediately after ice off. In May and extending through August, percent saturation moved above 100% for the top 10 m, indicating dominance of net photosynthesis over respiration in the mixed layer. The positive effect of net photosynthesis on percent saturation began to decline at the end of August, when oxygen production was overtaken by entrainment of water below the August thermocline as the mixed layer began to thicken. Even in June, at the height of light extinction caused by turbidity associated with inflowing surface water, the upper 7 m of the water column was exposed to solar irradiance ≥1% surface PAR, as necessary to support positive net photosynthesis. Under ice after mid-February and during full mixing, PAR deficiency suppressed net photosynthesis, as shown by persistent oxygen subsaturation of the upper water column (Fig. 12).

The decline of oxygen concentrations in deep water that began in June and extended into the middle of October supports an estimate of respiration. The rate of decline below 20 m depth was 0.02 mg O_2_ L^−1^ day^−1^; loss of oxygen under ice below 20 m showed a similar slope. These low oxygen depletion rates are consistent with dominance of refractory allochthonous DOC in the hypolimnion of the lake. Labile DOC released from phytoplankton was rapidly metabolized by bacteria in the epilimnion (Morris and Lewis, 1992).

### Phytoplankton community composition

Phytoplankton species from the mixed layer were counted on all sampling dates for 1992–2015 except 1996–1998 (missing). In 1981–1982, species were scored by abundance categories rather than species counts. Abundances for 1999–2016 were clustered as a moving average of 3 years for assessment of temporal abundance trends (Table III).

The cumulative record shows 252 species; the median number detected per year was 67. Community composition changed greatly over the study interval. The 10 most abundant species at the end of the study (2013–2015) included only one of the most abundant species for the beginning of the study (1992–1994, Table III). Strong contrasts applied also to genera. Only one genus from 1981 to 1982, prior to suppression of SRP, was among the top 10 group in 1992, following full implementation of tertiary treatment. Only 2 of the 10 most abundant genera in later years were among the 10 most abundant genera for the earliest years. From 1992 to 2015, the top 20 species included 5 that increased, 5 that decreased (all diatoms), 1 that showed consistently high rank (*Chrysochromulina parva*), 4 that both decreased and increased and 5 that showed no trend. Superimposed on these patterns was a great deal of interannual variation in rank among species, including brief strong declines or peaks (1–3 years) in abundance for a number of taxa.

**Fig. 13:**
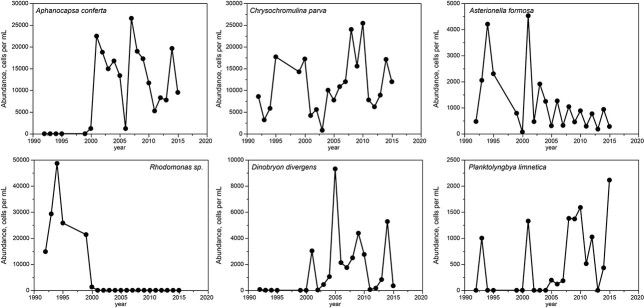
Numerical abundance (cells per mL) of phytoplankton taxa showing examples of species showing increase, decrease, and brief, dominant peaks or declines.

Figure 13 shows abundances as cell counts for selected taxa that show predominant trends. Absolute abundances are related in part to cell size, e.g. *Aphanocapsa* has a cell volume of ~1 μm^3^, whereas *Synedra* has a cell volume of ~500 μm^3^; the emphasis here is on change in abundance. While the community as a whole showed continuous change in the form of shifting dominance that deviated progressively from the initial composition, individual taxa showed wide interannual variation in abundance.

**Table III:** Top 20 abundant phytoplankton species of Lake Dillon for the latest interval (2013–2015), the earliest interval (1992–1994) and the entire time of sampling (1992–2015) as determined by abundance of cells

Taxon	Rank			Pattern
	2013–2015	1992–1994	1992–2015	
*Aphanothece clathrata* (C)	1	92	1	ID
*Chrysochromulina parva*	2	12	4	CH
*Aphanocapsa conferta*	3	93	7	I
*Dactylococcopsis* sp.	4	35	12	NT
*Synedra radians*	5	2	5	D
*Chlorella minutissima*	6	11	3	ID
*Discostella* (*Cyclotella*) *glomerata*	7	94	22	D
*Bitrichia ollula*	8	49	11	ID
*Cyanobium* sp.	9	95	13	I
*Dinobryon divergens* (S)	10	81	21	I
*Urosolenia* (*Rhizosolenia*) *eriensis* (C)	11	5	6	D
*Chroococcus dispersus*	12	96	44	NT
*Synechococcus capitatus* (C)	13	38	50	NT
*Planktolyngbya limnetica* (C)	14	39	33	NT
*Asterionella formosa* (C)	15	18	24	D
*Dinobryon cyclindricum* var. *alpinium* (S)	16	23	32	NT
*Synedra delicatissima* var. *angustissima*	17	22	26	D
*Kathablepharis* sp.	18	97	35	I
*Synedra rumpens* var. *familiaris* (C)	19	76	14	ID
*Cosmarium tenue* var. *depressum* (C)	20	98	98	I

Trends in rank over the full time span are scored as: I = increase, D = decrease, CH = consistently high, NT = no trend, ID = increase and decrease within the study interval. Letters in parentheses indicate abundance of genera in 1981–1982, prior to reduction of phytoplankton biomass by suppression of SRP: C = common, S = secondary (present but not abundant).

**Fig. 14:**
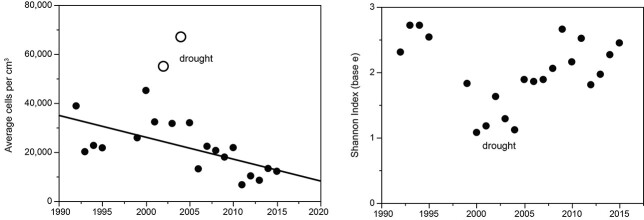
Left: Incremental change in annual average mean abundance of cells for phytoplankton in Lake Dillon, 1992–2015, *r*^2^ = 0.40, *P* = 0.003 (two drought years excluded). Right: Diversity of phytoplankton in Lake Dillon, 1992–2015.

Concurrent with constant interannual change in phytoplankton species composition was a decline in annual mean cell count (70%, Fig. 14). Over the same interval, chlorophyll declined (42%, Fig. 6), which indicates that mean cell size increased slightly with oligotrophication (~1.6×).

The Shannon diversity index showed mean diversity of 2.0 (base e) and no secular trend, but a strong suppression of diversity in years 2000–2005, was concurrent with a rise in dominance of cyanobacteria during drought. The diversity pattern for species was caused mainly by changes in evenness rather than species richness, which showed no trend and only moderate variance (mean 64 species per year, SD 11). At the division level, abundances declined over the entire sampling interval for most taxa: diatoms, chlorophytes, chrysophytes and cryptophytes, as reflected in decline of annual average cells per unit volume (Fig. 14). Haptophytes remained almost stable except for a dip in 2000–2005, when cyanophytes showed a peak. Dinophytes and euglenophytes were too scarce to be scored reliably.

### Zooplankton

Zooplankton populations of Lake Dillon were not sampled on an annual basis over the entire study interval. In 1981, a 12-month study of zooplankton in Lake Dillon showed that more than 99% of individuals in the zooplankton population consisted of four taxa: *Polyarthra vulgaris*, *Keratella cochlearis*, *Keratella quadrata* and *Diacyclops bicuspidatus* (Lewis *et al*., 1984). *Daphnia* and *Bosmina* were present, but in very small numbers. *Mysis diluviana*, sampled separately by Nelson (1981), also was present in significant abundance. *Mysis* was introduced in 1970 (Nesler, 1986); a population was present consistently after 1975 (mean for 1991–2009, 261 individuals per m^2^; Martinez *et al*., 2010). *Daphnia* (mostly *D. galeata*) had been abundant prior to 1970 (Nelson, 1981). Its subsequent scarcity indicates that *Mysis* caused an extreme reduction of *Daphnia* that persisted over the 35-year study interval, although a few years showed isolated sporadic occurrence of moderate abundance for *Daphnia* (e.g. 10–15 L^−1^  Martinez *et al*., 2010).

**Fig. 15:**
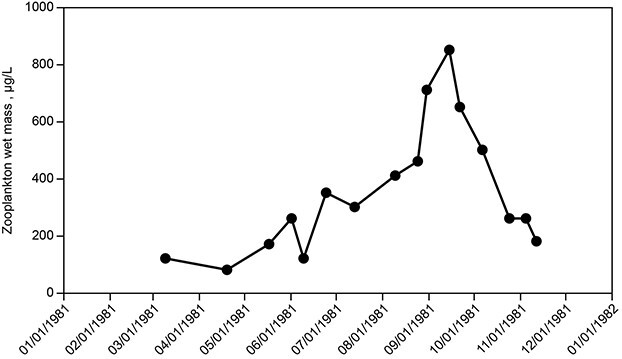
Wet mass abundance of total zooplankton in 1981; redrawn from Lewis et al. (1984).

The 1981 study showed a September peak for zooplankton biomass of ~800 μg L^−1^ wet mass within the top 20 m (Fig. 15). The median for the growing season (June–October) was ~400 μg L^−1^; winter populations often were small, but bursts of *Daphnia* near the ice cover occurred in multiple years. The median abundance of copepods during the growing season was ~30 L^−1^ (adults and copepodids; nauplii had nearly the same abundance but low biomass). Rotifers had a median numerical abundance of ~400 L^−1^ but contributed minimally to biomass (~10%).

Martinez *et al.* (2010) sampled zooplankton once per year in most years for 1991–2009. Johnson (personal communication 2017) also sampled zooplankton at least once annually in most years for 1993–2015. Both used a metered net with a mesh of 154 μm. These two sources report a median abundance of ~25 adult and subadult crustacean zooplankton L^−1^ (nauplii and rotifers not included; Martinez et al. had lower estimates, Johnson had higher estimates). *Bosmina* showed a gradual rise in population density to ~25 individuals L^−1^ in 2014 and 2015 (unpublished data, Brett Johnson 2017). A temporary abrupt surge in abundance of *Diacyclops* also occurred, resulting in a doubling of its abundance in 2014–2015. The combined results for all years, 1991–2015, were similar to those for 1981. Both of the multiyear sampling programs showed great variation interannually in zooplankton abundance (coefficient of variation ~50% across years). There were no long-term trends in total abundance, which was greatest in the middle of the 35-year interval.

## DISCUSSION

### Decline in phytoplankton biomass (chlorophyll)

An abrupt decline in chlorophyll for 1981–1984 coincided with reduced release of total P rich in SRP from treatment plants, but the second, more gradual, period of decline (1996–2016) was not coincident with decrease in total P loading (Figs 5 and 8). Factors other than P must be considered for both periods of biomass suppression, but other factors show no indication of potential for suppression of algal biomass over either of the two periods of decline. Nitrogen deficiency was not implicated at any time because loading of dissolved organic N (DIN) was not reduced when total P loading was reduced in 1981–1984, and because concentrations of DIN increased substantially subsequent to 1990 (Fig. 7). Factors other than nutrients that could explain decline in phytoplankton abundance, including hydrology, PAR and herbivory, failed to show a trend corresponding to decline in phytoplankton and had low capacity to suppress biomass in Lake Dillon. Water temperature did show secular warming (2.5°C mixed layer over 35 years, Lewis *et al*., 2019), but warming did not produce changes in mixed layer thickness or duration of ice cover, either of which could have affected phytoplankton (e.g. Berger *et al*., 2007). Significant changes in the mixed layer occur in response to warming of tropical lakes (Verburg *et al*., 2003; Hecky *et al*., 2010), which show substantial increase in density gradients with warming, whereas cold lakes show small changes in gradients in response to warming (e.g. Lewis *et al*., 2019). Increase in warmth of Lake Dillon can be assumed to have caused metabolic acceleration of algae (Reynolds, 2006; Domis *et al*., 2014), but this effect is minimal for lakes at high latitude or high elevation (Kraemer *et al*., 2017). For lakes generally, suppression of production or biomass that follows warming corresponds to warmth that exceeds an optimal temperature (Butterwick *et al*., 2005), which is unlikely for Lake Dillon given its low maximum temperatures (~17°C: Lewis *et al*., 2019).

In a study of maximum primary production per unit chlorophyll over 31 years of warming and oligotrophication for Lake Geneva, Tadonléké (2010) showed that warming was associated with increased production when total P was >22 μg/L and had a repressive effect at lower concentrations consistent with oligotrophication, but the generality of this pattern is not clear. Anneville *et al.* (2002) showed that oligotrophication was accompanied by an increase in algal biomass corresponding to a shift in species composition toward taxa with larger cells. Given that larger cells generally have lower metabolic rates (Banse, 1976; Reynolds, 2006), decline in *P*_max_ per unit chlorophyll could be consistent with a shift toward higher amounts of chla associated with larger cells in response to oligotrophication. For Lake Dillon, decline in phytoplankton biomass was concurrent with a modest increase in cell size, but phytoplankton biomass (chlorophyll) did not increase, it decreased.

An interpretation of P as the cause of chlorophyll suppression must be based on SRP loading of Lake Dillon rather than total P loading of Lake Dillon, given that total P loading showed no trend in loading after 1984. Phase 1, 1981–1984, showed suppression of 68% for SRP and was accompanied by chlorophyll suppression of 46% as a result of tertiary treatment of P in treatment plants. The strong effect of tertiary treatment on SRP is explained by the high ratio of SRP to total P in municipal effluent (Bali and Gueddari, 2015) and the strong response of SRP to tertiary treatment methods (Li and Brett, 2015).

After implementation of tertiary treatment, additional P recovery was developed primarily by attempts to control P transport from soil as erosion or infiltration (Phase 2, 1985–1995), which showed no measurable suppression of total P. Transport of P is a natural background component of loading but can be augmented by disturbance of soil or by facilitation of soil transport over impermeable surfaces (Dodds and Oakes, 2004), but P from this source is likely to have a low SRP:TP ratio (Sonzogni *et al*., 1982; Reynolds and Davies, 2001; Elkholm and Krogerus, 2003; Jarvie *et al*., 2006). Studies of the watershed showed that nonpoint sources of P other than septic systems accounted for only 70 kg/year, whereas municipal effluent prior to completion of tertiary treatment accounted for 825 kg/year (Fig. 4, Lewis *et al*., 1984).

Phase 3 (1996–2016) focused on control of P from septic systems, which was estimated from watershed studies to average 430 kg/year prior to installation of treatment plant connections to some of the oldest systems (Lewis *et al*., 1984). Recovery of P was estimated as 200 kg/year over Phase 3 and consisted primarily of dissolved P. Table II gives a direct estimate of the SRP component based on river runoff. The gradual reduction of SRP coincided with a gradual decrease in chlorophyll over the duration of Phase 3 (Fig. 8).

The match between changes in SRP loading over the three time spans and chlorophyll over the same spans supports the hypothesis that SRP controlled algal biomass in Lake Dillon.

### Fate of phytoplankton production

As shown by monitoring data, loss of biomass, as indicated by chlorophyll, from the mixed layer through spillway overflow removed only a small portion of net primary production from the lake. Availability of PAR in the mixing zone, which could affect production, showed no long-term trend. A third loss, sinking of phytoplankton cells, can be estimated from the literature. The largest of dominant taxa in Lake Dillon would sink at rates no greater than 1 m day^−1^ (Reynolds, 2006); the average sinking rate for phytoplankton typically is much lower. Reynolds (1989) showed that a sinking rate of 1 m day^−1^ for large cells corresponds to ~10% biomass loss per day from an epilimnion of 10 m thickness, which implies <5% per day loss of mixed algal cell types to sinking in Lake Dillon.

Grazing losses of phytoplankton can be estimated from zooplankton abundance. For 1981, prior to the decline in phytoplankton biomass, the measured mean biomass for zooplankton, June–October, was 400 μg L^−1^ wet mass (Lewis *et al*., 1984). The mean wet biomass of zooplankton populations for lakes with phytoplankton populations similar that of Dillon (6170 μg/L wet mass in 1981) can be estimated from the relationship between zooplankton and phytoplankton in a large group of lakes spanning a wide trophic range (McCauley and Kalff, 1981, Equation 1): 1380 μg/L. Thus, the abundance of zooplankton for Lake Dillon was 30% of the mean for lakes similar to lake Dillon; low relative abundance of zooplankton implies low grazing losses for phytoplankton.

A direct estimate of grazing rate can be based on characteristic filtration rates for lakes where grazing is dominated by copepods and cladocerans: at most 2000 mL mg^−1^ dry weight day^−1^ (Jassby and Goldman, 1974). Grazing at this rate would have cleared ~40% of net production per day. The low grazing rate in Lake Dillon explains why there was no suppression of phytoplankton biomass at the end of spring, in contrast with many lakes (Berger *et al*., 2007).

Loss rates for all mechanisms that can be estimated (grazing, outflow, sinking) account collectively for ~50% of primary production. Loss unaccounted for, quantified as a residual of documented losses, appears to be accounted for by cell death consisting of an unknown combination of programmed cell death (PCD or apoptosis, Bidle and Falkowski, 2004), mortality caused by cell-specific biological agents (e.g. viruses, bacteria) or environmentally induced death caused by lack of nutrients or other stresses (Franklin *et al*., 2006). These losses will be referred to here collectively as “endogenous mortality.”

A problem with quantifying a mortality mechanism through computation of a residual is the cumulation of error in all estimates that are antecedent to the residual (Jassby and Goldman, 1974). For Lake Dillon, however, losses that might have affected the estimate of a residual for loss of phytoplankton biomass were small. Grazing rate was low in Dillon as compared with other lakes of similar fertility, hydraulic loss of biomass from the mixed layer was suppressed by minimization of spillway overflow as a means of conserving the storage volume of water in the lake, and sinking was a very small loss relative to production. Because errors in estimating these three factors would not likely account for the size of the residual, endogenous mortality is strongly indicated.

Endogenous mortality has been observed (Lund *et al*., 1963) and quantified (Agusti *et al*., 2006) in field populations of lakes; losses of this type are especially evident in algal blooms and in laboratory cultures (Franklin *et al*., 2006). Lysis of phytoplankton cells appears to account for a high percentage of DOC release in oligotrophic marine waters (Agusti and Duarte, 2013). For Lake Dillon, the significance of endogenous mortality lies in consistent, significant loss of phytoplankton biomass in the mixed layer.

The closest comparison for Lake Dillon algal mortality is a study by Jassby and Goldman (1974) of Castle Lake, which had primary production very similar to that of Lake Dillon and showed epilimnetic endogenous mortality of 20–60%, as computed from all other losses. A difference between Castle Lake and Lake Dillon is grazing loss for phytoplankton, which was greater in Castle Lake than in Lake Dillon. For Lake Dillon, endogenous mortality may have been magnified by low loss of biomass to grazing.

Dynamics of phytoplankton below the mixed layer in Lake Dillon also show some unexpected features. The estimated endogenous loss of phytoplankton was ~50% within the epilimnion, but <10% in the hypolimnion (Fig. 9). As estimated by sinking rates and consistency of chlorophyll in the water column during stratification, the cells remained in the hypolimnion over deep water (e.g. 40 m) for at least a month. Cells between 10 and 20 m (metalimnion) showed significant losses, but cells at depth (hypolimnion) showed no significant mortality; their abundance in the absence of significant PAR was steady with depth during stratification. These cells originated from the mixed layer at a rate of ~0.6 μg L^−1^ chlorophyll per day. During the interval of descent toward the sediments, the cells appear to have been viable, as indicated by low phaeophytin corrections for chlorophyll measurements (typically 5–25% expressed as chla). The explanation for differences in apparent mortality between the deep water and surface populations of phytoplankton cells is not obvious, but the biochemical processes of algae in these two very different environments could be influenced by suppression of metabolic rate for cells descending to an environment that is aphotic and cold as compared with the environment of cells nearer to the surface. The fate of cells reaching the sediment surface is unknown, but they are likely the cause of moderate summer oxygen depletion (Fig. 12).

High endogenous mortality for phytoplankton in Lake Dillon would have provided a consistent source of labile organic matter in support of microbial metabolism. Morris and Lewis (1992) showed that microbial growth rates in Lake Dillon were far below optimal rates that could be achieved experimentally by enrichment *in situ*. Experimental enrichments showed that bacteria consistently had a strong growth response to addition of SRP, but almost never had a growth response to addition of labile organic matter. Release of dissolved organic matter through endogenous cell mortality combined with suppression of microbial growth by P deficiency, which could inhibit decomposition of released DOC, could account for the notable increase in DOC during the last part of the growing season (25%, Morris and Lewis, 1992).

### Changes in community composition of phytoplankton

Phytoplankton taxa or functionally defined groups can be classified to some degree according to their response to specific physical and chemical features of their environment (Lewis, 1986; Reynolds, 2006; Ptacnik *et al*., 2009). Classifications of this type may explain interannual change in composition of phytoplankton communities in response to climate change or change in trophic state. In Lake Dillon, only two environmental features, temperature and availability of SRP, showed long-term trends. There is presently no basis for predicting long-term trends in species composition in response to temperature changes of the magnitude observed in a cold lake such as Dillon (trend = 2.5°C over 35 years). The data suggest, however, that a slow decline in SRP loading, the likely cause of a persistent downward trend in chla for Lake Dillon, may have been also the cause of changes in phytoplankton community composition. This conclusion seems contrary to the accepted principle that changes in phytoplankton composition are explained by multiple, well-known factors under conditions of disequilibrium (Hutchinson, 1961; Interlandi and Kiham, 2001; Reynolds, 2006) through mechanisms of meteorological irregularity (Finger *et al*., 2013), chaos or spatial heterogeneity at large or small scales (Huisman and Weissing, 1999; Ptacnik *et al*., 2010). The Lake Dillon data indicate that SRP had a large, singular influence on community composition of phytoplankton over an extended interannual time scale.

Anneville *et al.* (2005) concluded that decline in P concentrations of European perialpine lakes caused synchronous changes in phytoplankton community composition, even though changes in phytoplankton biomass were negligible. Their data show that biomass was stabilized by shifts from small to large taxa in response to decreasing P, or by other factors including grazing (Jørgensen and  De Bernardi, 1998), whereas community composition did not show comparable resilience. Mechanisms accounting for differential viability related to cell size include capacity for P storage as it might affect resistance to strong SRP depletion, but data presented by Suttle *et al.* (1988) show no evidence of such an effect. In at least some lakes, some changes in composition may be predictable. For example, in large lakes of northern Europe, chrysophytes, pinnate diatoms and cyanobacteria sequentially dominate communities as algal abundance increases in response to nutrient enrichment (Ptacnik *et al*., 2008). Other interannual changes may be forecast on the basis of adaptations that are advantageous at low concentrations of bioavailable P, e.g. an increase in abundance of phagotrophic taxa (Jeppesen *et al*., 2002). Interannual changes in Lake Dillon do align temporally to some degree with taxonomy at the division level but much less coherently at the species level. Decline of diatoms and chlorophytes in Lake Dillon could have been a simple outcome of P deficiency, but simultaneous decline of cryptophytes and chrysophytes is not consistent with hypothetical advantages for phagotrophs when external SRP is scarce. A burst of cyanobacterial abundances in 2000–2005 coincided with drought that lowered water levels to an extreme extent. Wind generated currents reached the bottom of the water column, which increased concentrations of P in the water column, to which cyanobacteria apparently responded, but other taxa did not show clear responses except for haptophytes, which were temporarily suppressed. The most parsimonious explanation for secular change in species composition of Lake Dillon, which has an unusually narrow range of factors that could explain trends in phytoplankton, is response to gradual decline in SRP supply.

## GENERAL CONCLUSIONS

The long-term data record allowed detection and quantification of a long-term change in chlorophyll and its unique concurrence with a single environmental factor (SRP) and not with other factors that might cause such a change, thus simplifying the analysis of cause and effect and leading to a more secure conclusion than would have been possible on the basis of a short data record. A second and provocative aspect of the long-term data record, which would not have been evident from a short record, is the extensive change in phytoplankton composition over 20 years when the only continuous change in environmental factors that might cause extensive change in phytoplankton composition was the availability of SRP. No final conclusions are possible for this observation, but the implication is that progressive small changes in this single variable may cause large interannual changes in phytoplankton composition even though changes in phytoplankton composition typically are viewed on a seasonal basis, which focuses attention on the interacting roles of multiple variables that affect growth and decline of individual species populations in a specific growing season. The long-term perspective may be qualitatively different, as it was for Lake Dillon, where the importance of seasonal changes was superseded over decades by nonseasonal change in a single factor, SRP.
